# Common food additive carrageenan inhibits proglucagon expression and GLP-1 secretion by human enteroendocrine L-cells

**DOI:** 10.1038/s41387-024-00284-4

**Published:** 2024-05-16

**Authors:** Sumit Bhattacharyya, Alip Borthakur, Joanne K. Tobacman

**Affiliations:** 1 Department of Medicine, University of Illinois at Chicago, Chicago, IL USA; 2 Research, Jesse Brown VA Medical Center, Chicago, IL USA; 3 Department of Clinical & Translational Sciences, Marshall University, Huntington, WV USA

**Keywords:** Dietary carbohydrates, Obesity

## Abstract

Proglucagon mRNA expression and GLP-1 secretion by cultured human L-cells (NCI-H716) were inhibited following exposure to λ-carrageenan, a commonly used additive in processed foods. Carrageenan is composed of sulfated or unsulfated galactose residues linked in alternating alpha-1,3 and beta-1,4 bonds and resembles the endogenous sulfated glycosaminoglycans. However, carrageenan has unusual alpha-1,3-galactosidic bonds, which are not innate to human cells and are implicated in immune responses. Exposure to carrageenan predictably causes inflammation, and carrageenan impairs glucose tolerance and contributes to insulin resistance. When cultured human L-cells were deprived overnight of glucose and serum and then exposed to high glucose, 10% FBS, and λ-carrageenan (1 µg/ml) for 10 minutes, 1 h, and 24 h, mRNA expression of proglucagon and secretion of GLP-1 were significantly reduced, compared to control cells not exposed to carrageenan. mRNA expression of proglucagon by mouse L-cells (STC-1) was also significantly reduced and supports the findings in the human cells. Exposure of co-cultured human intestinal epithelial cells (LS174T) to the spent media of the carrageenan-treated L-cells led to a decline in mRNA expression of GLUT-2 at 24 h. These findings suggest that ingestion of carrageenan-containing processed foods may impair the production of GLP-1, counteract the effect of GLP-1 receptor agonists and induce secondary effects on intestinal epithelial cells.

## Introduction

Glucagon-like peptide (GLP)-1 is an incretin synthesized and secreted by enteroendocrine L-cells of the distal small intestine and colon. Production and secretion are modulated in response to both nutrients and non-nutrients in the gut lumen, including palmitic acid, oleic acid, and meat hydrolysates [1–4]. GLP-1 enhances glucose-stimulated insulin secretion from pancreatic islets, delays gastric emptying, and promotes satiety [2]. These effects lead to reduced food intake and weight loss and provide the foundation for the increasing clinical use of GLP-1 receptor agonists for the treatment of diabetes and obesity.

In this short communication, we present the unexpected finding that exposure of human L-cells to the common food additive carrageenan inhibited GLP-1 production. Carrageenan is a widely used natural product that improves the texture of processed foods due to its ability to bind with casein and other bioactive molecules, but it has no nutritional value. Anticipated daily intake in adults in the United States likely exceeds 100 mg/day and has been estimated to be between 18 and 40 mg/kg/day [5, 6]. Food safety regulatory authorities have repeatedly addressed the safety of carrageenan due to concerns about its use in infant formula and in a wide variety of food products [7]. Derived from red algae, carrageenan has been used in the scientific laboratory for decades since it predictably causes inflammation and can be used to test the effectiveness of anti-inflammatory pharmaceuticals and to identify inflammatory mediators. The three predominant forms of carrageenan (kappa, lambda, and iota) are composed of distinct sulfated disaccharides linked by alternating α-1,3 and β-1,4-glycosidic bonds. The alpha-gal bonds are not made by human cells and are associated with innate immune responses, including rejection of non-human organ transplants, tick bite illness, and meat allergy [8]. Experiments have shown that carrageenan exposure leads to the nuclear translocation of NF-κB, due to effects on reactive oxygen species and to interaction with toll-like receptor (TLR)-4 [9]. Glucose intolerance and insulin resistance occurred in mice exposed to oral carrageenan and were attributable to disruption of intracellular insulin signaling and activation of inflammatory pathways [10]. Other experiments indicated that carrageenan inhibited the responsiveness of the insulin receptor, due to effects on N-acetylgalactosamine-4-sulfatase (Arylsulfatase B, ARSB), chondroitin 4-sulfate, and galectin-3 interaction with the insulin receptor [11, 12]. In previous experiments, carrageenan did not have an independent effect on mouse weight or weight in patients with pre-diabetes on a no-carrageenan diet [13, 14], and the impact on incretins and mouse enteroendocrine cells was not tested.

To consider the impact of carrageenan exposure on GLP-1 production, experiments were performed in cultured human L-cells, mouse enteroendocrine cells, and human L-cells co-cultured with human intestinal epithelial cells (IEC). The effect of human L-cell secretion on IEC was addressed by the measurement of GLUT2 expression, which has been reported to be affected by GLP-1 [15].

## Materials and methods

Cell lines were procured and grown under the recommended conditions, including 37 °C and 5% CO_2_. The human intestinal L-cell line NCI-H716 (CCL-251, ATCC, Manassas, VA, USA) was originally isolated from the ascites fluid of a patient with colorectal adenocarcinoma [2]. LS 174 T cells (CL-188, ATCC) are colonic epithelial cells isolated from a patient with colorectal cancer. STC-1 cells (CRL-3254, ATCC) are an intestinal neuroendocrine cell line isolated from a mouse intestinal tumor [16].

The human and mouse L-cells were incubated overnight in low glucose medium without serum, then exposed to. high glucose (25 mM) DMEM with 10% FBS with or without λ-carrageenan (1 μg/ml; Sigma Aldrich, St. Louis MO, USA) for 10 minutes, 1 h, or 24 h. Spent media and cell homogenates were collected. GLP-1 protein levels in the spent media were measured by ELISA (Immuno-Biological Laboratories IBL-America, Minneapolis, MN, USA; #27784). mRNA levels of proglucagon, the precursor of GLP-1, were determined by QRT-PCR, using established procedures with the following primers:

proglucagon (human): left: 5′-CGTTCCCTTCAAGACACAGAGG-3′;

right: 5′-ACGCCTGGAGTCCAGATACTTG -3′;

proglucagon (mouse): left: 5′-CCTTCAAGACACAGAGGAGAACC-3′; and

right: 5′-CTGTAGTCGCTGGTGAATGTGC-3′.

The effect of carrageenan exposure on GLUT2 expression by the human intestinal epithelial cells was tested in an in vitro co-culture model. NCI-H716 human intestinal L-cells were grown on transwell inserts on top of a monolayer of human intestinal epithelial cells (IEC) (LS174T). The L-cells were treated with λ-carrageenan (1 μg/ml) for 10 min, 1 h, and 24 h, and the impact on mRNA expression of GLUT2 by the IEC was measured using the primers:

GLUT2 (human; NM_000340): left-5′-ATGTCAGTGGGACTTGTGCTGC-3′ and

right - 5′-AACTCAGCCACCATGAACCAGG-3′.

Statistics were performed with Microsoft Excel and PRIZM 9.5 software (GraphPad, Boston, MA, USA) using unpaired *t*-tests, two-tailed with unequal variance for all of the comparisons. Mean values and standard deviations of at least triplicate experiments are presented in the figures. *P* ≤ 0.05 is considered statistically significant.

## Results

**Fig. 1:**
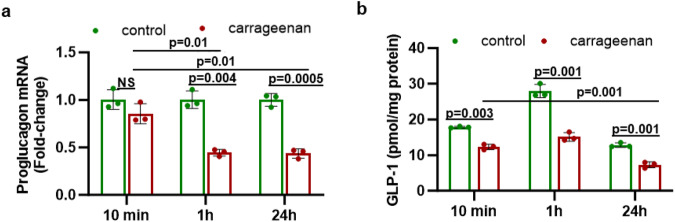
Carrageenan reduces GLP-1 production in enteroendocrine cells. **a** Following exposure to carrageenan (1 µg/ml) for 10 min, 1 h, and 24 h, mRNA expression of proglucagon (GLP-1) was measured in the human L-cell line. mRNA expression was significantly less at 1 h and 24 h following carrageenan than in the untreated controls (*p* = 0.004 and *p* = 0.0005, respectively; *n* = 3). In the carrageenan-treated cells, expression was 0.4-fold at 1 h and at 24 h, compared to the value at 10 min (*p* = 0.014, *p* = 0.010; *n* = 3), whereas there was no significant decline in the control cells. **b** GLP-1 secretion from the human L-cells was significantly less at all time points in the L-cells exposed to carrageenan than in the cells exposed to media without carrageenan (*p* = 0.003 at 10 min, *p* = 0.001 at 1 h, *p* = 0.001 at 24 h; *n* = 3). The secreted GLP-1 declined from 12.3 ± 0.7 at 10 min to 7.3 ± 0.8 pg/mg protein at 24 h following carrageenan exposure (*p* = 0.001, *n* = 3). All comparisons are by unpaired *t*-tests, two-tailed with unequal variance.

Following overnight deprivation, human L-cells were incubated with high glucose, serum, and carrageenan. The mRNA expression of proglucagon declined over time from 10 min to 24 h in the carrageenan-exposed cells, to 0.44-fold the level in the time-matched controls, which were exposed to high glucose and serum, but no carrageenan (*p* = NS at 10 min, *p* = 0.004 at 1 h; *p* = 0.0005 at 24 h; unpaired *t*-test, two-tailed, unequal variance; *n* = 3) (Fig. 1a). In the carrageenan-treated cells, mRNA expression was significantly less at 1 h and at 24 h than at 10 min (*p* = 0.014, *p* = 0.010), whereas there was no significant decline in the untreated control cells between 10 min and 24 h. Consistent with the decline in proglucagon expression, GLP-1 secretion by the L-cells into the media was significantly less than the time-matched control values following exposure to carrageenan (Fig. 1b). The secreted GLP-1 levels in the spent media following carrageenan vs. control were: 12.3 ± 0.7 vs. 17.8 ± 0.2 pmol/mg protein at 10 min (*p* = 0.003); 15.1 ± 1.2 vs. 27.9 ± 1.8 pmol/mg protein at 1 h (*p* = 0.001); and 7.3 ± 0.8 vs. 12.8 ± 0.6 pmol/mg protein at 24 h (*p* = 0.001).

Experiments were performed in mouse enteroendocrine cells to support the findings in the human cells, Proglucagon expression was also significantly less at 24 h following exposure to carrageenan (*p* < 0.01, unpaired *t*-test, two-tailed, unequal variance, *n* = 3), relative to the control with no carrageenan exposure (0.44 ± 0.09 fold-change compared to control value of 1.0 ± 0.18).

**Fig. 2:**
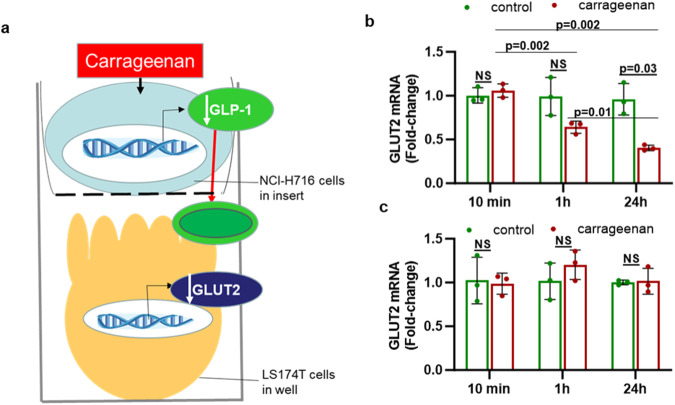
Impact of L-cell secretion on GLUT2 expression by co-cultured intestinal epithelial cells. **a** The schematic drawing shows the two-layer culture in which L-cells in filters were directly exposed to carrageenan. The product of the L-cells affecting the IEC may be GLP-1 or another secretory product. **b** GLUT-2 expression in the IEC at 1 h was 0.64-fold, and at 24 h, was 0.40-fold the value at 10 min, following co-culture with human L-cells exposed to carrageenan, compared to the value at 10 min (*p* = 0.002, *p* = 0.002, *n* = 3). At the same time, no-carrageenan control was significantly greater at 24 h (*p* = 0.03; *n* = 3). **c** Direct exposure of the IEC to carrageenan did not affect the mRNA expression of GLUT-2. All comparisons are by unpaired *t*-tests, two-tailed with unequal variance.

To test if products of the carrageenan-treated L-cells could affect transcription in the IEC, the mRNA expression of GLUT-2 was measured in co-cultured IEC. A monolayer of LS174T IEC was exposed to the spent media from carrageenan-treated L-cells grown on inserts, as depicted (Fig. 2a). Glucose Transporter (GLUT)2 expression in the IEC declined significantly (*p* = 0.03 at 24 h, unpaired *t*-test, two-tailed, unequal variance, *n* = 3) (Fig. 2b). In contrast, direct carrageenan treatment of the LS174T cells had no effect on the expression of GLUT2 (*p* > 0.05) (Fig. 2c).

These data indicate that carrageenan exposure inhibited the expression and secretion of GLP-1 by human intestinal L-cells. Mouse L-cell expression of proglucagon was also reduced. Also, spent media from the carrageenan-treated human L-cells inhibited the GLUT2 expression in co-cultured IEC, indicating the potential for significant paracrine effects of L-cell products, including GLP-1, on the surrounding IEC. GLUT-2 was not modified by direct exposure to carrageenan.

## Discussion

Current interest in the effects of incretin-based therapy, including GLP-1 receptor agonists and dipeptidyl peptidase-4 inhibitors, is intense due to effects on weight loss, as well as diabetes treatment. The experiments presented in this report suggest that consumption of carrageenan in processed foods may act to reduce the endogenous secretion of GLP-1 and the effectiveness of these therapeutic agents. Previous work indicated no significant effect on weight in carrageenan-treated mice or in individuals on a carrageenan-elimination diet for 12 weeks [13, 14]. However, these study findings suggest that carrageenan exposure may affect weight over the long-term by the impact on GLP-1 production and by inhibition of response to treatment by GLP-1 receptor agonists, thereby limiting weight loss.

Effects of carrageenan on transcription may be attributable to its inhibition of the enzyme N-acetylgalactosamine-4-sulfatase (Arylsulfatase B; ARSB) [17]. ARSB mediates transcriptional events through effects on chondroitin 4-sulfate, galectin-3, and SHP2, and these effects may contribute to the current findings [18]. Additional effects of carrageenan may be mediated by secondary effects of GLP-1 or other L-cell secretory products by paracrine effects on neighboring cells, affecting transporters such as GLUT2. GLUT-2, by effects on glucose transport, may participate in the regulation of responses to ambient glucose concentration [19]. Further studies are required to define the interactions and possible feedback mechanisms involving these vital mediators of glucose homeostasis.
